# A Framework for Implementing Integrated HIV and Non-Communicable Disease Care at Primary Health Care Facilities in Southern Africa

**DOI:** 10.5334/ijic.8944

**Published:** 2025-07-09

**Authors:** Maureen Moyo-Chilufya, Tennyson Mgutshini, Charles Hongoro, Alfred Musekiwa

**Affiliations:** 1 School of Health Systems and Public Health, Faculty of Health Sciences, University of Pretoria, Pretoria, South Africa; 2 University of South Africa, College of Graduate Studies, Pretoria, South Africa; 3 Human Sciences Research Council, Pretoria, South Africa

**Keywords:** framework, HIV, integrated, noncommunicable diseases, primary healthcare, Southern Africa

## Abstract

**Introduction:**

Comorbidities of HIV/AIDS and non-communicable diseases (NCDs) are increasingly prevalent, affecting up to 30% of individuals living with HIV/AIDS, particularly in Sub-Saharan Africa. Conventional approaches that treat NCDs separately from HIV/AIDS care have been deemed inefficient, highlighting the need for integrated models. This study aims to develop a framework for integrating NCD care into HIV programs at primary healthcare facilities in Southern Africa.

**Methods:**

As part of a broader study examining the burden, extent and cost of HIV/NCD integration we employed a modified ‘Best fit’ framework synthesis method. Thematic analysis was the primary method of analysis used to inform the framework.

**Results:**

The study expanded on existing framework themes related to effective team-working, organizational leadership, patient-centered care, community/patient/provider partnerships. It introduced seven additional themes: country specific NCD prioritization, multi stakeholder partnerships, healthcare worker mental wellbeing, unified health information systems, with enhanced privacy, establishing costing databases for HIV/NCD integrated care, robust monitoring and evaluation mechanisms, and opportunities for regional coordination.

**Conclusion:**

Improving existing frameworks for integrating HIV/NCD care is feasible by leveraging established HIV care platforms. These enhancements can support more efficient holistic healthcare delivery across primary healthcare facilities in Southern Africa.

## Background

Human immune deficiency virus infection (HIV) and non-communicable diseases (NCDs) remain important public health problems in Southern Africa, which is the home of the majority of people living with HIV (PLHIV), globally [1].

The prevalence of HIV is highest in Eswatini (25.9%) [2], followed by Lesotho (19.3%) [3], Botswana (16.4%) [4], and South Africa (13.9%) [5]. Furthermore, South Africa has the highest number of people living with HIV at 8.5 million [5]. All the Southern African countries with a high burden of HIV also have a high proportion of NCD related deaths. For example, Eswatini, Lesotho, Botswana and South Africa have NCD related death proportions of 37%, 32%, 46% and 51%, respectively [6].

Despite countries having attained the UNAIDS 95-95-95 targets [1], gains made in HIV care may be lost due to the rising burden of NCDs, as indicated by a recent systematic review with the burden of NCDs among PLHIV, ranging from 1% to 30% [7]. Criticism has been directed towards the conventional approach of treating NCDs in PLHIV separately from HIV/AIDS care, deeming it clinically inefficient. Consequently, the integration of NCD care into the HIV care programs emerges as a novel treatment paradigm providing benefits of leveraging on the well-established HIV care programs in the Southern African region to strengthen health systems for provision of NCD care at primary health care (PHC) facilities [8,9,10,11,12,13]. Notably, the establishment of an integrated HIV/NCD care programme provides an opportunity to expand these services to the rest of the population, and thereby fostering universal health coverage (UHC) [10].

Guided by the above noted priority, the current study sought to address the pressing need for integrating HIV and NCD care in PHC facilities in Southern African countries. Our objective was to formulate a comprehensive framework that facilitates the seamless integration of HIV and NCD care, thereby enhancing the effectiveness of health systems in these resource-constrained settings.

## Methods

**Figure 1:**
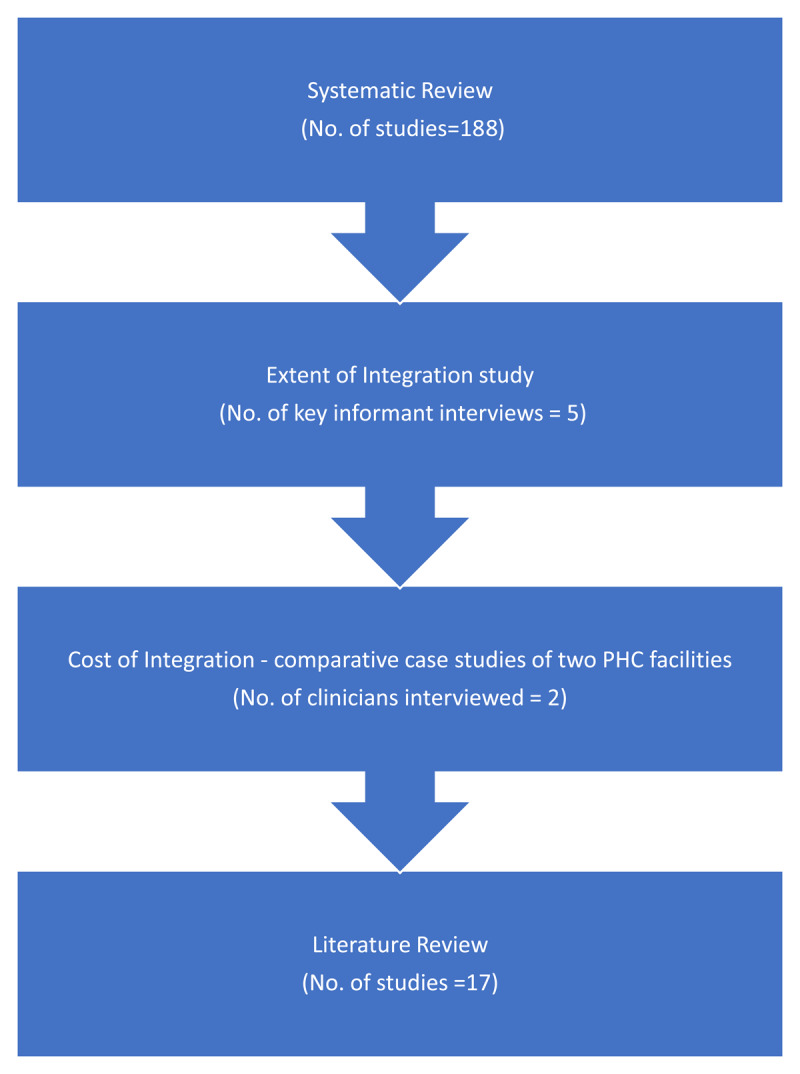
The four data collection approaches utilised to build the framework.

The methodology adopted for the study was made up of four empirical and theoretical data collection approaches (Figure 1).

We utilised a modified version of the “Best Fit” framework synthesis (BFFS) method described by Carroll C et al [14]. This method involves both framework and thematic analysis techniques to compile the synthesis and is usually accompanied by a systematic review of literature. We utilised the following steps of the BFFS method: 1) Formulated the review question 2) Conducted a literature review to identify existing conceptual or theoretical frameworks for integrating NCDs into HIV care at PHC facilities or general chronic disease care integration into PHC services. 3) The results of the review were analysed to construct an *a priori* framework 4) Coded the study evidence against the *a priori* framework 5) Interpreted any data that could not be placed within the framework using inductive, thematic analysis 6) Developed a framework incorporating both the *a priori* and new themes identified from the primary research, including insights from three prior studies. 7) Further thematic analysis resulted in the creation of a developed framework.

**Figure 2:**
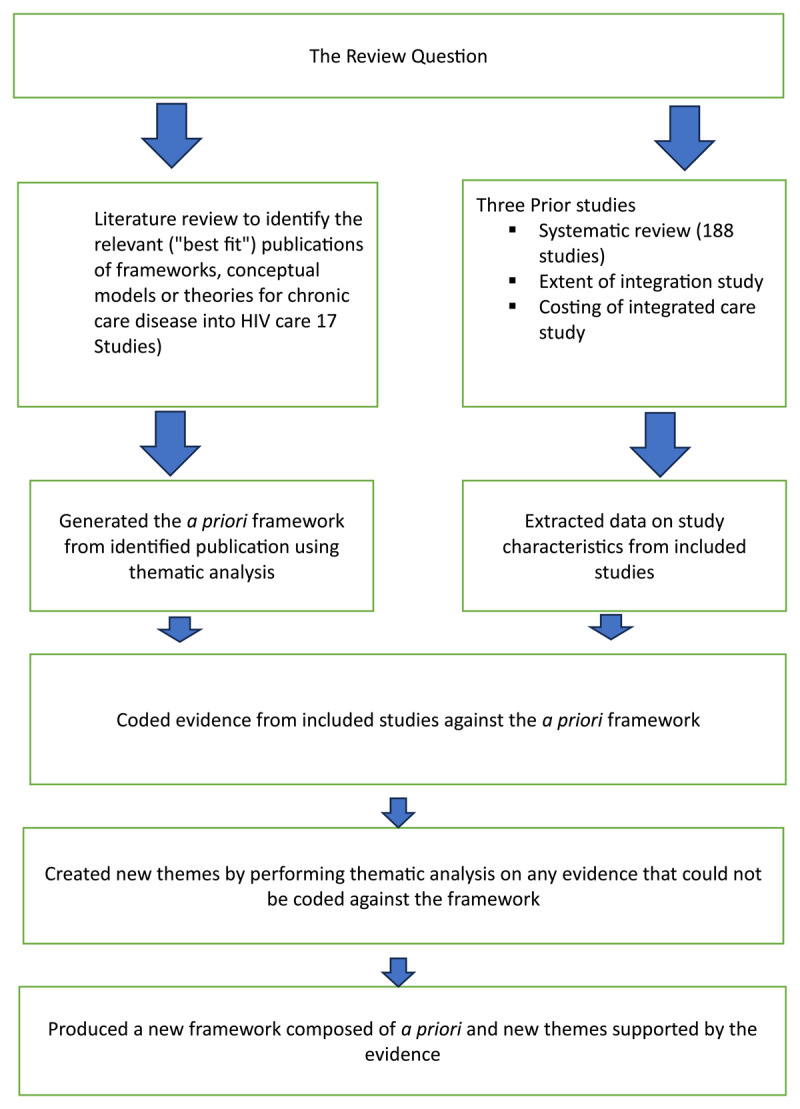
Summary of the framework development process.

The selected theoretical framework was reduced to its key elements and variables which formed the themes of the *a priori* framework. Evidence from the identified studies from the search were coded along with the primary data collected from three prior studies. Figure 2 offers a summative overview of the framework development process.

In our study we utilised a non-systematic literature search because we wanted to inform the framework from insights obtained from our three prior studies, that included a systematic review, hence the authors were familiar with the current publications. The three preceding studies included a systematic review on the burden of non-communicable diseases among PLHIV [7], an extent of integration study (unpublished data) and costing of providing an integrated HIV/NCD care service as case studies at PHC facilities in Southern Africa [15]. During the extent of integration study, we interviewed national HIV programme managers that shared their perspectives and insights on the extent of integrated HIV/NCD care within their respective countries. We obtained informed consent from the managers before the interview. Details of the interview are reported in a separate article. Furthermore, based on the costing study [15], health facility managers provided insights into the integrated process of caring for patients with both HIV and NCDs.

We conducted the literature search on Pubmed and Google scholar. Authors were familiar with keywords utilised for the search from prior studies conducted for the integration of HIV/NCD care in Southern Africa. The main search terms used were “Integrated”, “framework”, “HIV”, “AIDS”, “NCDs”, “Diabetes”, “cardiovascular disease”, “hypertension,” “depression”, “chronic respiratory disease” and Africa”. The inclusion criteria of studies were HIV/NCD framework studies, studies on HIV/NCD integration, studies that were relevant to SSA, not limited by date of publication and language. We excluded studies that did not include HIV/NCD care and studies that were irrelevant to SSA.

The main selection criteria focussed on the theme of integrated HIV care with any of the four major NCDs and depression in Sub Saharan Africa. The literature search utilised the snowball search strategy based on keywords, concepts and themes in order to complement information from our previous studies. Selected studies included both peer reviewed publications and grey literature.

Themes forming the *a priori* framework were identified from the selected study [16], with a conceptual framework on the integration of NCDs into primary health care services. The evidence from the selected studies was coded along with the primary data collected from three preceding studies. The analysis and coding was done manually using the *a priori* themes. We selected 17 publications that informed the developed framework.

### Involvement of people with lived experience

The first author possesses lived experience as defined by the International Journal of Integrated Care (IJIC). Her specific involvement is described in the author contributions’ section.

### Ethics

Ethical review for this study was obtained from the University of Pretoria, Faculty of Health Sciences, Ethics Review Committee (Ref number: 591/2021).

### Results

The study aimed to develop a framework for the integration of NCD care into HIV/AIDS care programmes in Southern Africa.

From the literature review, we selected a framework on chronic disease care integration into PHC services developed by Harrison and Jordan, 2022 [16]. This framework was based on the chronic care model(CCM) [17], and the innovative care for chronic conditions framework (ICCCM) [18]. The *a priori* themes are shown in Table 1 as articulated by Harrison and Jordan, 2022 [16] People living with HIV, without complications in SSA, are cared for at PHC level. For this reason, we utilised this framework to develop a framework for integrating NCDs into HIV care. We isolated the themes from this framework to determine the *a priori* themes. From the 17 other selected articles, we coded for themes to either provide more evidence for the existing themes or to develop new themes for the developed framework. Among the articles that were used to inform the developed framework are the WHO Afro framework 2017 [8] and the WHO implementation guidance on integrating the prevention and control of NCDs in HIV/AIDS, tuberculosis, and sexual and reproductive health programmes [10].

**Table 1:** *A priori* framework themes for integrated chronic disease care into primary healthcare services [16].


*A PRIORI* FRAMEWORK THEMES

1. Effective team-working to deliver continuity and coordinated proactive care

Organizational leadership, culture, and mechanisms to promote quality and safety

Equipped health care teams to deliver evidence-based patient-centred care

Empowerment and support of patients for self-management and prevention

Use of data collection systems to facilitate effective care and follow-up

Community partnerships to promote awareness, mobilize resources and support health service provision

Improving patient access to chronic disease care

Task shifting

Clinical mentoring

Stigma and confidentiality

Patient provider partnerships


## Identification of new themes

### Identifying priority NCDs for each country for integration into HIV care services

This theme was derived from the WHO guidance document [10] and supported by Kintu et al [11]. Furthermore, a recent systematic review showed that despite numerous studies on the burden of NCDs among PLHIV in SSA, data was not available for most of the countries in the region [7]. The majority of studies are conducted in a few select countries signifying the need for more research to be conducted in underrepresented countries in order to identify which NCDs should be prioritized in their respective HIV/NCD programs. As stated by WHO, there is a need for context specific findings per country [10]. Upon identification of the priority NCDs, the need to adapt WHO guidelines to context specific guidelines arises [8].

### Stakeholder Partnerships for HIV/NCD integrated care at PHC

The *a priori* framework alludes to the importance of patient/provider, and community/provider partnerships. Additionally, WHO [10] included private/public partnerships as important in the delivery of an integrated NCD service. Resultantly, we reframed these themes to create “stakeholder partnerships” as important for HIV/NCD integrated care service delivery at PHC facilities. These partnerships could allow for resource mobilisation, for example formation of drug clubs, with the support of the community, to supplement drug shortages [19]. The private/public partnerships in addition to resource mobilisation, could be ideal platforms to engage corporates that are directly involved in industry that is involved with products that are known to promote NCDs in the general population, such as the tobacco, food and alcohol industries [10]. With all stakeholders engaged, prevention and care for NCDs among PLHIV is likely to improve and strengthen the health systems.

### Supporting the mental wellbeing of healthcare workers

The increase in workload due to an integrated HIV/NCD service has been reported in a recent scoping review [12]. Although task shifting has been identified as remedial to staff shortages and assisting with handling workload, it is not uncommon for healthcare workers (HCWs) to experience burnout [20]. Some of the proposed solutions to support HCWs from the a priori themes highlighted the need to incentivise HCWs, both financially and non-financially [8,10,16]. Additionally, we found a need to go a step further to prioritize the mental wellbeing of HCWs [21].

### Establishment of health information systems for PLHIV that have HIV and NCD data in one place and to improve privacy and confidentiality of patients

There is a need for integrating the health information systems to have patient health records for PLHIV in one place for HIV and NCDs. From the costing study we found that HIV data was kept separate from the NCD data. In addition to providing efficiency for accessing patient health records, integrating health information systems could also aid in mitigating stigma at the PHC facilities as all patients carry similar coloured and barcoded patient cards. It was noted from the extent of integration study (unpublished) that some countries have disease-specific colour coded patient cards. This system may foster stigma, unlike the use of patient barcodes that are not identifiable with a particular disease. This theme also speaks to the a priori component of improving data collection systems to facilitate effective delivery of health care [8,10,16].

### Establishment of a costing database for HIV/NCD integrated care

Due to the dearth of costing and cost effectiveness data for HIV/NCD integrated care, creation of costing databases [13] for PHC facilities would improve the availability of such data.

In our case study, we utilised the activity-based costing method [22] to estimate the cost of HIV/NCD integration at two PHC clinics in South Africa, as case studies, a method that can be used routinely for costing of the integrated programme, ensuring availability of costing data that can be used for budgeting, resource mobilisation, and research.

### Improved monitoring and evaluation processes that inform progress made in the HIV/NCD integrated programme

Including NCD care performance indicators into the HIV monitoring and evaluation program will help visualise progress made with respective performance indicators such as patient outcomes for both NCDs and HIV [8,10]. In a similar way that HIV programme performance indicators are well designed and utilised, there is a need to improve the monitoring and evaluation process of the HIV/NCD integrated service at PHC level.

### Regional Coordination Opportunities

As observed during the COVID-19 pandemic, Africa needs to utilise the regional economic communities to strengthen their health systems and fight future pandemics more effectively [23]. For example, the Southern African Development Community (SADC) can be utilised to implement integrated NCD care and facilitate coordinated efforts, with the inclusion of patients crossing borders [24] and requiring access to both HIV and NCD drugs. Well-coordinated regional efforts could prepare the existing health systems for future pandemics, having designed efficient systems and in turn produced a more resiliently healthy population. Additionally, regional efforts could aid in resource mobilisation and provide opportunities for negotiated pricing for essential drugs with manufacturers.

**Figure 3:**
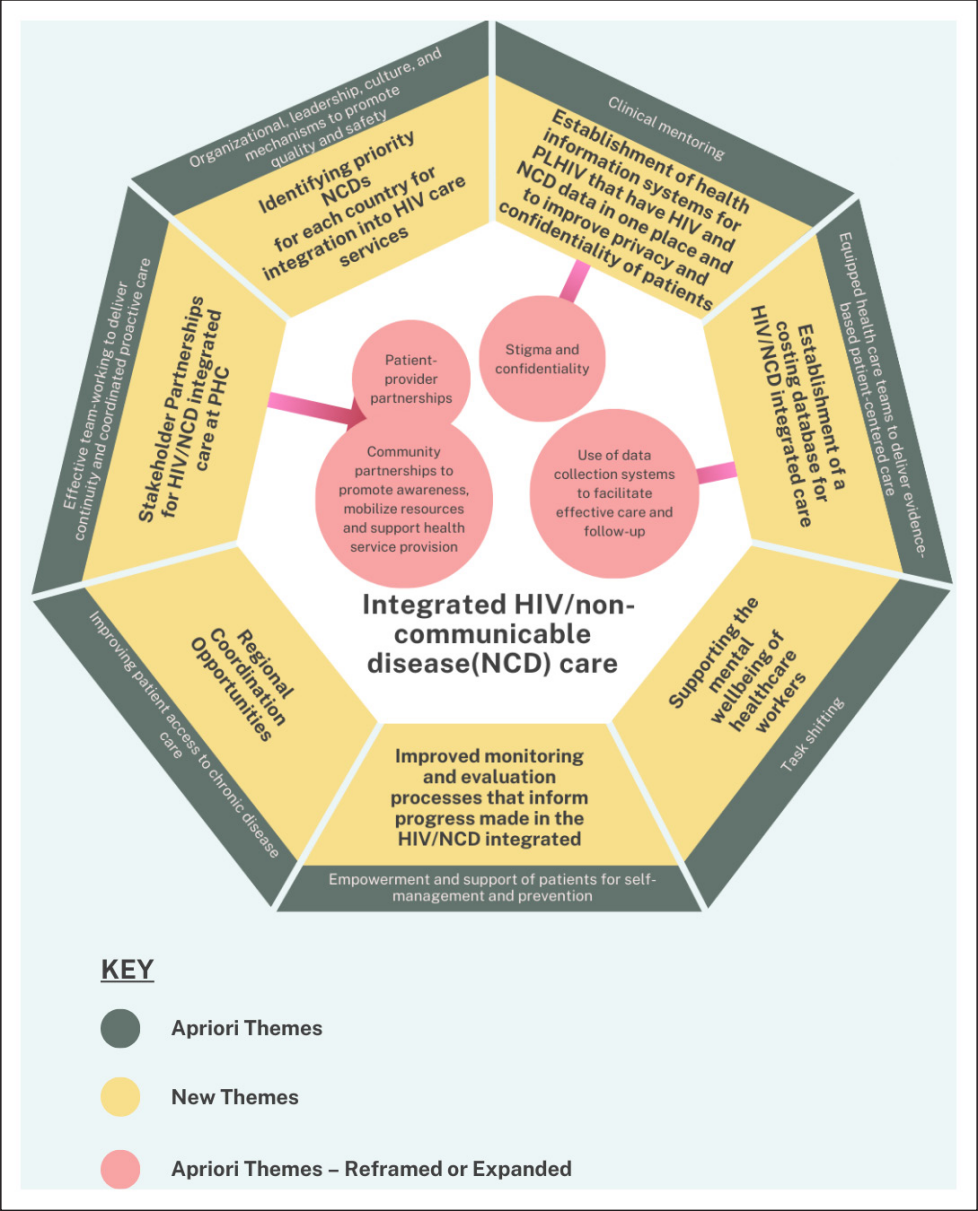
A summary of a framework for implementing HIV and non-communicable disease care at primary health care facilities in Southern Africa.

We show a summary of the developed framework for implementing integrated HIV and NCD care at primary health care facilities in Southern Africa Figure 3.

## Discussion

This study employed a modified method of the BFFS [14] to develop a conceptual framework for the integration of NCD care into the HIV care programs at PHC facilities in under resourced Southern African settings. Our framework development, is based on the model of integration that incorporates NCD care into the existing HIV care platforms at PHC facilities [9] with a focus on prioritizing cardiovascular diseases, diabetes, cancers and chronic respiratory diseases, alongside mental illnesses.

The *a priori* framework [16] was patient-centred and thus focused on the micro (patient interaction) level integration.

Building upon this foundation, the updated framework expands its scope to encompass the meso (public healthcare) level and the macro (community) level considerations. While maintaining its patient-centred approach, this framework extends beyond HIV and diabetes integration to include all the major NCDs and depression. The developed framework is grounded in international recommendations, particularly from the WHO Afro region [8] and the recent WHO guidelines [10] document on integrating prevention and control of NCDs with HIV/AIDS programme. These guidelines inform the theoretical foundation of the framework, ensuring its relevance and applicability to regional best practices, while tailoring the approach to local contexts.

To effectively implement integrated HIV/NCD care services at the PHC level, its essential for countries to first identify priority NCDs and concentrate efforts on these, particularly, those most relevant to their population. Given resource constraints, prioritisation becomes imperative as an initial step.

Stakeholder partnerships are pivotal in the successful implementation of integrated services. Collaborative efforts involving all community stakeholders enhances the likelihood of success. This theme extends beyond traditional patient/provider and community/provider partnerships to include the private/public partnerships, emphasizing the importance of community-wide engagement. Such partnerships create an enabling environment for integrating HIV/NCD care and enhance the likelihood of successful implementation by ensuring collective ownership and support across various sectors.

An important aspect of the framework is the recognition of the challenges faced by health care workers [20], particularly in settings marked by staff shortages, excessive workloads and other challenges. Therefore, it is crucial for leadership to prioritise their mental wellbeing, enabling them to deliver professional and compassionate care to clients. By prioritizing the well-being of the workforce [21], the framework advocates for the implementation of policies and support systems that enable healthcare workers to perform their roles effectively.

Integration of health information systems is also paramount for effective service delivery at the PHC facility. Leveraging technology allows for centralized data storage and visualization, potentially improving privacy and confidentiality by eliminating the need for disease specific identifiable patient cards or files, while improving service efficiency. Additionally, the framework calls for the establishment of costing databases [15] at PHC facilities, as a means to address the paucity of cost data for integrated HIV and NCD care. By adopting methods such as activity based costing, healthcare systems can more accurately assess the financial requirements and sustainability of integrated services. Understanding costs is crucial for effective budget planning and resource mobilisation needed to support the integrated health services. While the prior costing study did not include a cost-effective analysis, the comparison between integrated care costs and standalone services suggests that integrated care could be more cost-effective [15].

Lastly, the framework underscores the importance of robust monitoring and evaluation mechanisms to assess the progress and impact of integrated HIV/NCD services. The successful transition from theoretical to practical implementation requires continuous assessment, and the collaboration of regional economic centres such as SADC, is critical for fostering cross-border cooperation and support.

In summary, the developed framework is a multi-dimensional model that advocates for the implementation of integrating NCDs into existing HIV care programs, with a focus on, prioritizing NCDs, strengthening stakeholder partnerships, improving the mental well-being of healthcare workers, unifying health information systems and addressing the financial and logistical challenges inherent in these integration efforts. By promoting regional collaboration and enhancing monitoring and evaluation efforts, the framework seeks to ensure the sustainability and effectiveness of integrated HIV and NCD healthcare delivery systems in resource-limited settings.

### Limitations of the study

Although our study provides valuable insights, it is important to acknowledge that it had some limitations. Firstly, our search was not systematic, potentially resulting in the possibility of omission of informative publications. The BFFS method relies on available primary studies and there is generally a paucity of primary studies on frameworks for integrated HIV/NCD care at primary healthcare facilities in LMICs. Additionally, our key informants contributing to the development of the framework via our prior studies were from four countries within Southern Africa, namely, Eswatini, Mozambique, Zambia and Zimbabwe. Therefore, the generalisability of our results to the entire Southern African region may be limited.

### Implications for future research and practice

Our findings underscored the necessity of assessing the burden of NCDs for PLHIV in most countries of Southern Africa to tailor context specific prioritization strategies in these under-resourced settings. In addition to training, it is crucial to offer incentives to healthcare workers who assume added responsibilities in integrated care, prioritizing both their workload and mental wellbeing. Despite observed progress in patient screening and care integration, there are gaps regarding unified health data systems for NCD and HIV data at primary healthcare level. We also observed the need for costing databases for integrated HIV/NCD care services at PHC facilities. There is a need for further research on cost effective strategies of integrating NCD care into HIV care programmes for PLHIV and expanding care to the general population, as a step towards universal health coverage.
